# Exercise Training Effects on Inflammatory Gene Expression in White Adipose Tissue of Young Mice

**DOI:** 10.1155/2012/767953

**Published:** 2012-12-24

**Authors:** Tracy Baynard, Victoria J. Vieira-Potter, Rudy J. Valentine, Jeffrey A. Woods

**Affiliations:** ^1^Department of Kinesiology and Nutrition, University of Illinois at Chicago, 1919 W. Taylor Street, MC-517, Chicago, IL 60612, USA; ^2^Department of Nutrition and Exercise Physiology, University of Missouri, Columbia, MO 65211, USA; ^3^Department of Kinesiology and Community Health, University of Illinois at Urbana-Champaign, Urbana, IL 61801, USA

## Abstract

We aimed to determine the effects of 6 wks of exercise on inflammatory markers in mice concomitantly fed either high-fat (HF) or normal chow (NC) diets in young mice. C57BL/6 mice were randomized into (*n* = 10/group) an NC/sedentary (NC/SED), NC/exercise (NC/EX), HF/SED, and HF/EX groups. Treadmill exercise was performed 5 d/wk at 12 m/min, with 12% grade for 40 min/d. Liver triglycerides and gene expression of F4/80, MCP-1, TNF-**α**, leptin, and VEGF in visceral white adipose were determined. NC groups had lower body weights after 6 wks versus the HF groups (22.8 ± 0.2 versus 25.7 ± 0.4 g) (*P* < 0.0001). F4/80 gene expression (indicator of macrophage infiltration) and liver triglycerides were greatest amongst the HF/SED group, with no differences between the remaining groups. VEGF (indicator of angiogenesis) was greatest in the HF/EX versus the other 3 groups (*P* < 0.05). Exposure of an HF diet in sedentary young mice increased visceral adipose depots and liver triglycerides versus an NC diet. Exercise training while on the HF diet protected against hepatic steatosis and possibly macrophage infiltration within white adipose tissue. This suggests that moderate exercise while on an HF diet can offer some level of protection early on in the development of obesity.

## 1. Introduction 

 Recent reports have highlighted white adipose tissue (WAT) as one of the primary contributors to the chronic low-grade inflammation commonly observed in obesity and related complications (e.g., type 2 diabetes, metabolic syndrome) [1]. As WAT expands, it has been hypothesized that microhypoxia results in adipocyte stress and, ultimately, death via aponecrosis [2]. Adipocyte death and extravasation of fat into the extracellular space provide strong signals for macrophage recruitment and inflammation within WAT [2]. Several important cytokines are implicated in these processes, including monocyte chemoattractant protein-1 (MCP-1), tumor necrosis factor-*α* (TNF-*α*), interleukin-6 (IL-6), and vascular endothelial growth factor (VEGF). Chemokines, including MCP-1, are in part responsible for recruiting macrophages to the area of inflammation, which further contribute to local WAT inflammation by releasing their own host of cytokines [3]. VEGF has been shown to be necessary for WAT growth in order to provide microvascularization as the WAT expands in order to prevent microhypoxia and resulting WAT inflammation [4].

 Only recently have studies examined the effects of lifestyle modification on reducing WAT inflammation [5]. We have demonstrated that after establishing obesity (6 wks of high-fat diet (HF)), groups of mice placed on either a 12-wk treadmill moderate exercise program or low-fat diet (LF), or combination intervention were protected from the 3–5-fold increase in inflammatory markers in WAT as compared with HF diet sedentary mice [6]. This has been further supported in a separate strain of mice [7]. Additionally, groups of mice underwent the same treatments in a shorter time period (6 wks of treadmill training and/or LF/HF), with no differences found in these same markers [6]. Taken together, those findings suggest that a longer-term intervention (i.e., 12 versus 6 wks) is necessary to protect against the inflammatory damage induced by long-term HF diet. This has been further substantiated by Kawanishi et al. [8], whereby mice exercised for 16 wks on an HF diet experienced reductions in macrophage and inflammatory markers without marked improvements in body weight or adipose tissue accumulation. Whether or not exercise during the initial consumption of HF diet could have *prevented* or *attenuated *the deleterious inflammatory effects of HF diet could not be determined from these studies. With obesity on the rise in young people [9], it is important to understand if exercise plays an important role in either preventing or minimizing complications associated with obesity during this vulnerable age.

 Therefore, the purpose of this study was to determine the effects of *concomitant* HF (45% fat) feeding and 6 wks of exercise training on adipose tissue inflammation in young mice compared to young mice on a chow (17% fat) diet. Utilizing a 4-arm model (e.g., HF-sedentary (HF-SED), HF-exercised (HF-EX), normal chow-sedentary (NC-SED), NC-exercised (NC-EX)), we hypothesized that diet-induced weight gain would be observed in both HF diet groups with a lesser degree found in the HF-EX group. Further, we hypothesized that inflammatory markers would be upregulated with the HF, yet exercise training would attenuate these markers in the WAT. 

## 2. Methods

### 2.1. Design

Mice were randomly assigned to one of 4 groups. These groups included (1) HF-SED, (2) HF-EX, (3) NC-SED, and (4) NC-EX. Groups underwent simultaneous diet modification and exercise training for 6 wks, after which mice were euthanized for tissue collection and analyses. 

### 2.2. Animals and Diets

C57BL/6 male mice from Jackson Laboratories (Bar Harbor, ME) arrived at 4 wks of age and were individually housed (*n* = 40). Following 2 wks of acclimatization, 20 of the mice were randomized to an HF diet for 6 wks, with the remaining mice continuing their chow diet. The HF diet was a 45% fat diet purchased from Research Diets (New Brunswick, NJ), with the chow diet provided by Harlan (17% fat). Food and water were provided *ad libitum*. Body weights and food disappearance were monitored weekly throughout the study. Animals were kept on a reverse light-dark cycle, with lights on at 2100 h and off at 0900 h. The animal protocol was approved by the University of Illinois at Urbana-Champaign's Institutional Animal Care and Use Committee, and animal health and care guidelines were strictly followed.

### 2.3. Treadmill Training

The mice were treadmill trained during their dark cycle for 40 min/d at 12 m/min, with 12% grade, 5 d/wk for 6 wks. Training occurred on a motorized treadmill (Jog-a-Dog; Ottawa Lake, MI) between 0900 and 1030 h, with a gradual increase in duration over the first week of training, so that all runners were trained at the intended intensity by the 6-7th session. This training intensity has been shown to equate to ~65%–70% of peak aerobic capacity [10]. Negative reinforcement was not utilized in the training of these mice. All of the runners in this study complied with the training. All of the sedentary animals were exposed to the same treadmill noise and handling as the runners in attempt to control for stress associated with treadmill exposure. Treadmill training was employed over wheel running in order to accurately deliver a specific volume of exercise at moderate intensity.

### 2.4. Sacrifice

Animals were euthanized 48 h following their last exercise session in a fasted state (12 h fast) using CO_2_ inhalation.

### 2.5. Hepatic Triglycerides

After sacrifice, livers were extracted, weighed, frozen on dry ice, and stored at −80°C until analysis. Liver tissue (50–200 mg) was homogenized for 7–10 min in 4 mL of isopropanol with a polytron disrupter, with the resulting homogenate centrifuged at 4,500 rpm for 10 min. The supernatant (5 *μ*L) was used to determined triglyceride (TRG) content (L-type) (Wako Diagnostics; Richmond, VA).

### 2.6. Real-Time Quantitative PCR

Epididymal fat pads were immediately harvested after euthanization, weighed, aliquoted, frozen on dry ice, and stored at −80°C until analysis. RNeasy Mini Kits (Qiagen; Valencia, CA) were used for determination of total RNA. RNA was quantified and purity assessed using a NanoDrop spectrophotometer (Thermo Scientific; Wilmington, DE). AffinityScript (Stratagene, Agilent Technologies; Palo Alto, CA) reverse transcription was used to create cDNA from the mRNA according to the manufacturer instructions. Lastly, an Mx3000P Real-Time PCR machine was used to perform PCR using Brilliant II SYBR Green Master Mix kits (Stratagen, Agilent Technologies; Palo Alto, CA). The thermal protocols consisted of (1) denaturing for 10 min at 95°C, (2) 40 cycles of 95°C for 30 s, and (3) annealing at 60°C for 1 min and 72°C for 30 s. The housekeeping gene was glyceraldehydes-3-phosphate dehydrogenase (GAPDH). The ΔΔCT method was used to represent the data relative to GAPDH expression as fold change from NC-SED. All duplicate *C*_*T*_ (critical threshold) values were within 0.5 units from each other, and no group differences in *C*_*T*_ were observed for GAPDH among the runs of PCR (*P* values ranged 0.60–0.96 among the different primers). Primers were designed online by Integrated DNA Technologies (http://www.idtdna.com/) and verified by the National Center for Biotechnology Information (NCBI) “BLAST” feature (http://blast.ncbi.nlm.nih.gov/Blast.cgi). Primers were then purchased from Eurofins MWG Operon (Huntsville, AL). Specific details regarding the primers can be obtained upon request.

### 2.7. Glucose

Whole blood was obtained from the mice immediately prior to sacrifice using the retro-orbital eye bleed technique. Blood was centrifuged for 15 min at 2500 rpm at 4°C with the plasma aliquoted and stored at −80°C. Plasma glucose was later determined on a YSI 2300 Glucose analyzer (Yellow Springs Inc., Yellow Springs, OH) using the glucose oxidase method. 

### 2.8. Statistics

A one-way analysis of variance (ANOVA) with repeated measures was used to test for changes in body weight throughout the 6-week study period between groups (week × group). A two-way ANOVA (diet × exercise) was used to test for differences in baseline body weight, final body weight, change in weight, fat pad weight, liver weight, liver TRG, glucose concentrations, food intake, and WAT mRNA gene expression data. Following a significant finding, LSD post hoc tests were used to determine specifically where the differences were. The epididymal fat pad weight/body weight ratio was used as a covariate in determining the effects of epididymal adiposity on the exercise effects. Analyses were performed using SPSS v.17 (SPSS; Chicago, IL). Data are presented as mean ± SE. Alpha was set at a *P* value of ≤0.05.

## 3. Results

### 3.1. Body Weight, Fat Pad Weight, and Caloric Intake

**Figure 1:**
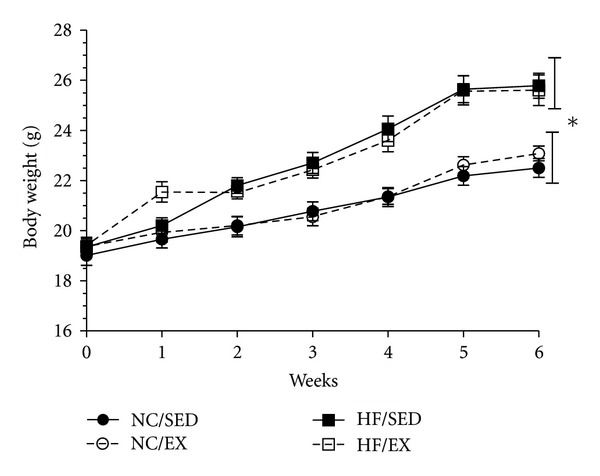
Weekly body weights over the course of 6 wks while mice were exercise trained (EX) or sedentary (SED) and on either normal chow or high-fat diets (NC, HF). *Closed circles*: NC/SED; *open Circles*: NC/EX; *closed Squares*: HF/SED; *open Squares*: HF/EX. *Diet effect only (*P* < 0.05). High-fat (45%) feeding resulted in overall greater body weights versus NC groups. Data are mean ± SEM. *n* = 10/grp.

Figure 1 depicts the body weights of the mice over the 6-wk study period. No differences in body weight were observed between the 4 randomized groups at baseline. Differences in body weight were evident beginning at week 1 and continuing through to 6 wks, with the HF diet groups having greater body weights than the NC groups (*P* < 0.05). Surprisingly, no differences in body weight between EX and SED, HF or NC, groups were observed at any time point. Epididymal fat pad weight expressed in absolute grams or relative to body weight were lowest in the two NC groups, with the HF-EX group having the highest values, followed by the HF-SED (*P* < 0.05) (Table 1). Lastly, total estimated caloric intake over the 6-wk period was only different between the two diet groups (*P* < 0.05), with no effect of exercise.

### 3.2. Liver Weight, Liver Triglycerides, and Fasting Plasma Glucose

**Figure 2:**
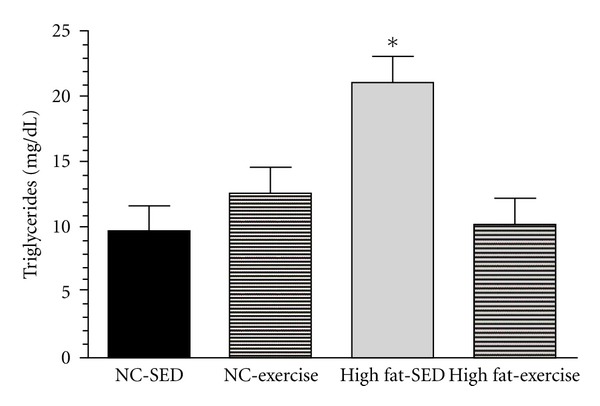
Liver triglycerides concentrations among the 4 experimental groups. High fat/sedentary (HF/SED) had the greatest triglyceride content compared to the 3 remaining groups (*P < 0.0001) (low fat/SED, LF/exercise (LF/EX), and HF/EX), with no differences among these three groups.

No group differences were observed in liver weights (Table 1). Liver triglycerides were greater in the HF-SED group compared to all other groups (*P* < 0.0001), with no differences observed between the 3 remaining groups (Figure 2). Plasma glucose was not different between groups at the end of 6 wks (Table 1). 

### 3.3. Gene Expression in WAT

**Figure 3:**
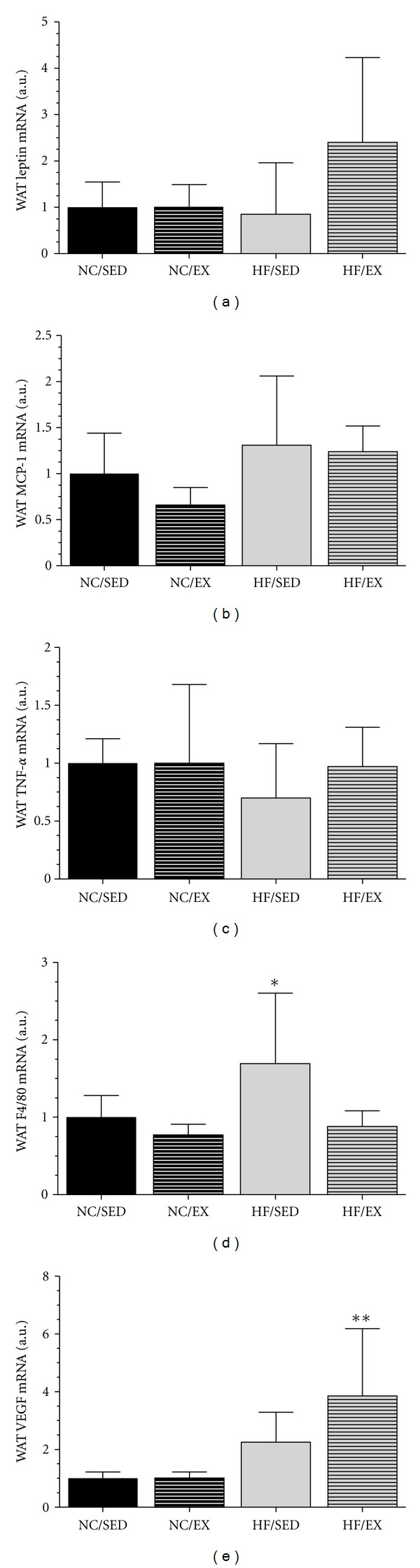
Leptin (a), monocyte chemoattractant protein-1 (MCP-1) (b), tumor necrosis-*α* (TNF-*α*) (c), F4/80 (d), and vascular endothelial growth factor (VEGF) (e) gene expression represented as fold change over the normal chow/sedentary (NC/SED) group. *High fat/sedentary (HF/SED) had greater F4/80 gene expression versus remaining 3 groups (*P* < 0.05). **HF/exercise (EX) expressed greater VEGF versus the other groups (*P* < 0.05). Data normalized to GAPDH, with no differences in GAPDH between groups. *n* = 8-9/grp.

No difference between groups was observed for leptin, MCP-1, and TNF-*α* mRNA gene expression data (Figures 3(a)–3(c)). However, F4/80 mRNA gene expression was greatest in the HF-SED group (*P* < 0.05), with no differences in the remaining 3 groups (Figure 3(d)). Lastly, VEGF mRNA gene expression was greatest in the HF-EX group (*P* < 0.05), again with no differences among the 3 other groups (Figure 3(e)). No group differences in control gene (GAPDH) were observed.

## 4. Discussion

The main findings from this study were that exercise did not attenuate weight gain in either the chow or HF diet groups but did appear to “normalize” liver triglyceride content in the HF diet group compared to that of the chow groups. Further, exercise training did not alter several potent inflammatory markers (i.e., TNF-alpha, MCP-1, and leptin) but did appear to have an effect within the HF diet group for F4/80 (lowered) and VEGF (increased) expression. These data suggest that in the face of a fatty “insult,” before the onset of established obesity, moderate exercise training exerts beneficial effects at two locations known to be potent contributors to chronic inflammation—the liver and WAT. 

Not surprisingly, the mice fed the HF diet gained more weight compared to the NC-fed mice. This body weight difference was evident as early as one week into the HF diet feeding. The exercise training protocol did not attenuate the HF diet-induced weight gain, nor did it reduce body weight gain in NC-fed mice. The fact that exercise training did not attenuate weight gain in either group suggests that the intensity was indeed moderate and coaligns with human data suggesting that moderate-intensity exercise alone may not be an effective weight-loss strategy [11]. Our protocol was unlike voluntary wheel running protocols, which generally result in higher exercise duration (e.g., 3–8 km/night) and are known to induce a significant caloric deficit resulting in weight loss [12]. Moreover, the mice were fed *ad libitum* throughout this study, and training did not increase caloric intake within each diet. Again, this validates the moderate intensity level of the exercise intervention, as exercise protocols that are strenuous enough to produce weight loss generally result in an increase in voluntary energy intake [12, 13]. 

Although the moderate exercise training protocol used did not result in a reduction in weight gain, it was sufficient to normalize HF diet-induced hepatic steatosis. This finding corroborates other work that has shown exercise to be an effective means to reduce hepatic steatosis [6, 14]. These current findings suggest that exercise training, even in the absence of weight loss, prevents HF diet-induced hepatic steatosis, a characteristic of metabolic dysfunction, in a young mouse model obesity. Although exercise did not reduce weight gain, regional body fat deposition was altered, as indicated by a training-associated reduction in epididymal fat mass in the mice on HF diet. As adiposity is closely related to the metabolic syndrome, which includes hepatic steatosis, future research will need to address whether the protective effect of exercise on liver triglyceride accumulation is mediated through a reduction in visceral fat *per se* or a reduction in adipose tissue inflammation. In support of this, Johnson et al. [15] demonstrated that aerobic exercise reduced hepatic and visceral fat in obese adults without reducing body weight. Further, Kanda et al. [16] suggest an MCP-1-dependent connection between adipose tissue macrophage infiltration and hepatic steatosis.

 While we did not observe any group differences in leptin, MCP-1, and TNF-*α*, all of which are considered to be important markers of inflammation in obesity, we did observe elevated F4/80 mRNA in WAT only in the HF-SED group compared to HF-EX and chow-fed groups. This suggests that macrophage infiltration (as indicated by F4/80 mRNA) precedes upregulation of inflammatory markers stemming from the WAT early on in the development of obesity. More importantly, moderate exercise training normalized F4/80 mRNA, comparable to the NC groups. It is also possible that the macrophages were not producing TNF-*α*, contributing to our lack of findings on that front. However, our data supports that reported by Strissel et al. [17, 18]. In their time course studies, they reported that metabolic disturbances were evident after 6 wks of an HF diet (60% fat) and that activated macrophage markers are also present at 6 and 8 wks (F4/80 and CD11c) [17], while interferon-*γ* (IFN-*γ*) and interleukin (IL)-12p40 were not significantly altered by the HF diet after 4 or 8 wks of feeding [17] nor were TNF-*α* or MCP-1 after 4 or 8 wks [18]. Our data supports the lack of an inflammatory response within epididymal adipose tissue after approximately 6 wks of exposure to an HF diet, while macrophage infiltration is suggested with the increase in F4/80.

Kawanishi et al. [8] also reported no weight loss or adipose tissue loss with a 5 d/wk, treadmill training protocol (60 min/d) following a 16-wk intervention, yet TNF-*α* and F4/80 gene expression were rescued by the training in their HF diet mice (60% HF diet) along with a decrease in CD11c (M1 macrophage marker). While we also report an attenuation in F4/80 in the HF-EX group, along with an increase in VEGF, it is important to note that our study is one of the first to report such findings in a group of young mice exposed to an HF diet for a relatively short period of time while undergoing concomitant exercise training. This demonstrates that this time period of high-fat exposure is an important period for investigation, and efforts in this area may help improve health outcomes. Our most salient finding is that moderate exercise training early on in the development of obesity “normalizes” macrophage infiltration gene expression in white adipose tissue, even in the absence of weight loss. This may be particularly important in relation to youth obesity. The mechanism for the “anti-inflammatory” effect of exercise needs further investigation.

 Recent work has shown that hypoxia, resulting from hypertrophied adipocytes with the progression of obesity, is causatively related to the inflammatory milieu that develops in obese adipose tissue [19–22]. Moreover, diminished vascular expansion in the adipose tissue with obesity has been suggested to be a possible trigger of the hypoxia that ensues. Interestingly, recent work has shown that exercise training increases endothelial cell density and VEGF gene expression in the adipose tissue of rats fed chow diet [23]. We report here that exercise training also increases VEGF gene expression in HF fed young mice. One possibility is that exercise training facilitates an adipose tissue compensatory response, such that capillary density increases with an expansion of adipose tissue mass. This would then prevent the adipocyte hypertrophy-associated reduction in oxygenation, resulting in hypoxia (and inflammation). Oxygenation assessment of the adipose tissue was beyond the scope of this current study, but the hypothesis that exercise training facilitates the angiogenic response in adipose tissue to prevent hypoxia and the resultant exacerbation of inflammation needs further investigation. 

 Although the results of this study are suggestive of a weight-loss independent anti-inflammatory effect of exercise that occurs early on in the development of obesity, there are limitations of this study that need to be considered as the data are evaluated. Insulin resistance was not assessed in this study, which is an important barometer of metabolic health and is known to affect even young individuals during the progression of obesity [24]. Thus, future studies should assess the effect of exercise training on insulin sensitivity in young mice on HF diet. Other studies have examined the effects of exercise on insulin sensitivity while on an HF diet in rodent models [25–27], with these studies generally using older models or not specifically stating the age of the mice or rats; therefore, using younger models is warranted. Second, relative gene expression levels do not always correspond to protein expression; thus, future studies should measure protein levels of inflammatory markers and quantify immune cells in order to more adequately assess the inflammatory profile of WAT with exercise training. Similarly, in order to more precisely assess the angiogenic potential of WAT, future planned studies will measure capillary density of WAT after exercise training, in addition to a better characterization of macrophage infiltration. 

 In conclusion, in the absence of inflammation in the intraabdominal WAT depot, as assessed by gene expression levels of inflammatory markers, moderate exercise training concomitant with HF feeding protected young mice against excess liver triglyceride accumulation. Moreover, exercise was associated with reduced gene expression of F4/80 (indicative of macrophage infiltration) and increased expression of the angiogenic marker VEGF within the WAT. These changes were observed without exercise-induced differences in body weight, suggesting that the effect of exercise is independent of body weight loss. The mechanism may be related to a slight exercise-mediated reduction in epididymal fat pad mass, as observed in the HF-EX group. 

## Figures and Tables

**Table 1:** Descriptive characteristics.

Variable	Normal chow-sedentary	Normal chow-exercise	High fat-sedentary	High fat-exercise
Epididymal pad (g)	0.36 ± 0.03^a^	0.38 ± 0.03^a^	0.88 ± 0.08^b^	0.65 ± 0.08^c^
Epididymal pad/body wt (%)	1.66 ± 0.19^a^	1.64 ± 0.19^a^	3.38 ± 0.20^b^	2.50 ± 0.18^c^
Total caloric intake (kcal)	394 ± 8^a^	401 ± 10^a^	442 ± 10^b^	440 ± 9^b^
Liver (g)	0.93 ± 0.03	1.03 ± 0.06	0.87 ± 0.04	0.85 ± 0.03
Fasting glucose (mmol/L)	9.8 ± 0.6	10.3 ± 0.4	10.0 ± 0.7	9.8 ± 1.0

Letters that are different signify group differences (*P* < 0.001).

Liver and Glucose values did not exhibit any group differences.
